# Population-wide impacts of aspirin, statins, and metformin use on prostate cancer incidence and mortality

**DOI:** 10.1038/s41598-021-95764-3

**Published:** 2021-08-09

**Authors:** Hye Yeon Koo, Su-Min Jeong, Mi Hee Cho, Sohyun Chun, Dong Wook Shin, Jinsung Park

**Affiliations:** 1 Health Promotion Center, CHA Bundang Medical Center, Seongnam, Republic of Korea; 2 Department of Family Medicine, Samsung Medical Center, Sungkyunkwan University School of Medicine, 81 Irwon-Ro, Gangnam-gu, Seoul, 06351 Republic of Korea; 3 Samsung C&T Medical Clinic, Kangbuk Samsung Hospital, Sungkyunkwan University School of Medicine, Seoul, Republic of Korea; 4 International Healthcare Center, Samsung Medical Center, Seoul, Republic of Korea; 5 Supportive Care Center, Samsung Comprehensive Cancer Center, 81 Irwon-Ro, Gangnam-gu, Seoul, 06351 Republic of Korea; 6 Department of Digital Health, SAIHST, Sungkyunkwan University, Seoul, Republic of Korea; 7 Department of Clinical Research Design and Evaluation, SAIHST, Sungkyunkwan University, Seoul, Republic of Korea; 8 Department of Urology, Uijeongbu Eulji Medical Center, Eulji University, Uijeongbu-si, Republic of Korea

**Keywords:** Epidemiology, Cancer epidemiology, Cancer prevention, Cancer, Urological cancer, Prostate cancer

## Abstract

We evaluated the association between aspirin, statins, and metformin use and prostate cancer (PC) incidence and mortality using a large population-based dataset. 388,760 men who participated in national health screening program in Korea during 2002–2003 were observed from 2004 to 2013. Hazard ratios of aspirin, statins, and metformin use for PC incidence and PC mortality were calculated with adjustment for simultaneous drug use. Cumulative use of each drug was inserted as time-dependent variable with 2-year time windows. Aspirin use ≥ 1.5 year (per 2-year) was associated with borderline decrease in PC mortality when compared to non-users (adjusted hazard ratio [aHR] 0.71, 95% confidence interval [CI] 0.50–1.02). Statins use was not associated with either PC incidence or PC mortality. Metformin ever-use was associated with decreased PC incidence compared with non-diabetics (aHR 0.86, 95% CI 0.77–0.96). Diabetics who were not using metformin or using low cumulative doses had higher PC mortality than non-diabetics (aHR 2.01, 95% CI 1.44–2.81, and aHR 1.70, 95% CI 1.07–2.69, respectively). However, subjects with higher cumulative doses of metformin did not show increased PC mortality. In conclusion, metformin use was associated with lower PC incidence. Use of aspirin and that of metformin among diabetic patients were associated with lower PC mortality.

## Introduction

Prostate cancer (PC) is the second most frequently diagnosed cancer in men worldwide, with an estimated 1.3 million new cases in 2018^1^. In Korea, the incidence of PC has been increasing over the past few decades^2^; the age-standardized incidence rate of PC was 28.2 (per 100,000) in 2016, which is similar to the global incidence of 29.3 in 2018^1,2^. Despite a significant decrease in the PC mortality rate in the past 20 years, PC remains the leading cause of cancer deaths among men in 46 countries, and the seventh leading cause of cancer deaths in Korean men^1,3^.

Recent studies have suggested that certain pharmacologic agents like aspirin, statins, or metformin may prevent cancer development and metastasis^4–8^. These medications are commonly used to treat non-cancer-related medical conditions and have shown favorable long-term adverse effects profile in the general population, which makes them attractive candidates as cancer chemopreventive agents^4^.

Several studies have reported that regular use of aspirin reduces the risk of aggressive PC and PC recurrence^9,10^. Although the association between aspirin and PC mortality varies across studies, extended post-diagnosis use of aspirin^11^ and high dose aspirin^12^ has been found to be associated with reduced PC-specific mortality. Metformin has also been shown to be associated with reduced risk of PC in some studies^13,14^. One study reported that increasing duration of metformin use was associated with a decreased incidence of PC^13^, and another population-based study reported that cumulative duration of metformin exposure was associated with decreased PC mortality^15^. Statins use was also reported to be associated with a general risk reduction and, specifically with advanced PC^16,17^, as well as reduced PC mortality^18,19^.

However, some studies have reported conflicting results with regard to use of these medications and their associations with PC incidence and mortality^20–22^. Furthermore, the earlier studies had several limitations. First, most previous reports did not examine the effect of cumulative medication use^9–12^: Some have investigated the effect of treatment duration or prescribed dose^11,12^, but few study exists regarding the cumulative dose of drugs. Rates of aspirin, metformin, and statins use have increased over the past decades^23–25^, but non-adherence to these medications is also common^26–28^. To clarify the chemoprotective effect of medication, cumulative medication exposure should be taken into account^29^. Second, another important aspect to be considered is the simultaneous use of medications. Because type 2 diabetes mellitus, dyslipidemia, and other cardiovascular diseases share several risk factors and tend to co-exist, aspirin, statins, and metformin are often prescribed concomitantly^30,31^. Therefore, the independent effect of each medication should be analyzed taking into account potential interactions between co-prescribed medications. However, the majority of previous studies did not adjust for the concomitant use of other medications. Several studies made adjustments for the ever-use of other medications, but the cumulative doses of these medications were not considered^11–13,18,19^. Third, representative data on the Asian population are lacking.

In this study, we evaluated the association between aspirin, statins, and metformin use and PC incidence and PC-specific mortality using a large population-based dataset.

## Materials and methods

### Study population and data source

We used the Korean National Health Insurance (KNHI) database for this study. In Korea, the National Health Insurance Service is a mandatory social insurance that covers the entire population of South Korea, and its database contains information on sociodemographic factors, clinical diagnosis, health care usage, pharmacy claims data, and vital status. KNHI also provides biennial health screening targeted towards cardiovascular risk factors, and this screening includes health behaviors assessment, anthropometric measurements, and laboratory testing for blood glucose and lipid levels.

KNHI provides a sample database for research purposes, and we pooled three sample cohorts of KNHI for our analysis: (1) the National Health Insurance Service (NHIS)-Senior Cohort, (2) the NHIS-Health Screening Cohort (NHIS-HealS), and (3) the NHIS-National Sample Cohort (NHIS-NSC). The NHIS-Senior cohort consists of 10% randomly selected samples of the Korean population aged 60 years or above. NHIS-HealS dataset comprises 10% random samples of Koreans aged 40 to 79 years who have participated in the national health screening program. Finally, The NHIS-NSC cohort consists of 2.2% random samples of the total Korean population. Since KNHI provides de-identified data for these cohorts, we were not able to rule out overlap among the three cohorts.

**Figure 1:**
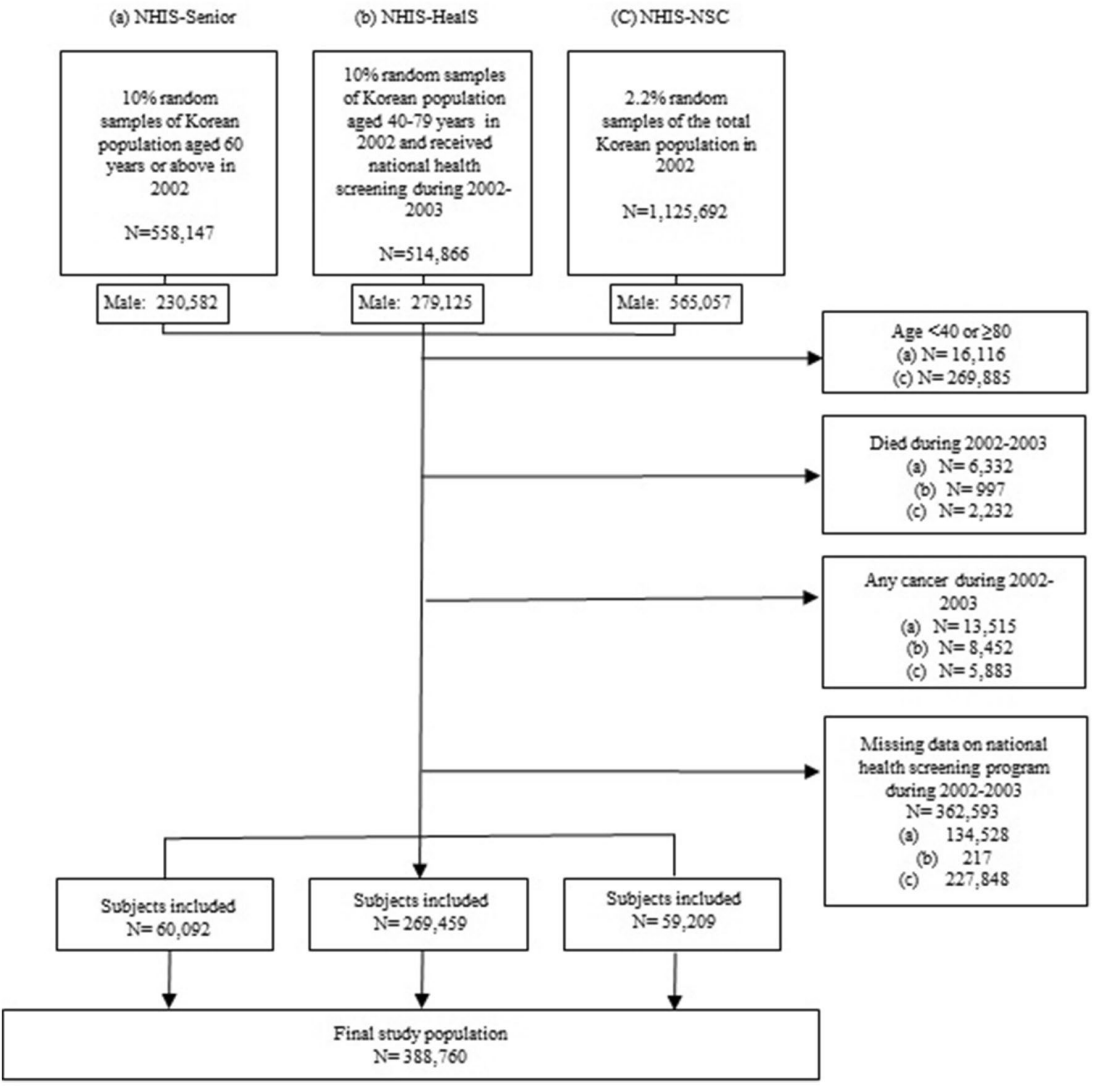
Summary of patient recruitment.

Study population comprised those individuals who are alive as of January 1, 2002. We used a 2-year time window for time-dependent analysis (Supplementary Fig. S1), and the first 2 years (2002–2003) were regarded as the baseline period. We included male subjects aged 40–80 years who had undergone national health screening during 2002–2003 (n = 788,763) to obtain information on smoking, alcohol consumption, and body mass index (BMI). Of the 788,763 eligible subjects, those who died (n = 9561) or had a diagnosis code of any cancer (n = 27,850) during the first 2 years (2002–2003), as well as those with missing data for variables from the national health screening program during 2002–2003 (n = 362,593), were excluded. The final study population comprised 388,760 subjects (Fig. 1).

### Study outcomes and follow-up

The main outcomes of this study were incidence of PC and PC-specific mortality between January 1, 2004 and December 31, 2013. A PC cancer case was defined as a patient who visited a health facility with the ICD-10 diagnosis code C61 and received any active treatment for PC (surgery, radiation therapy, or androgen deprivation therapy) claimed in the KNHIS data. Patients who underwent watchful waiting or active surveillance were not included as PC cases because they could not be accurately identified from the database^32^. PC-specific death case was ascertained through the Korean National Death Registry data based on a C61 code as a cause of death among all eligible participants. The date of the event was defined as the first date of the C61 code diagnosis. Patients were followed-up for analyses of PC incidence until diagnosis of PC, death from any causes, or December 31, 2013, whichever came first. For PC-specific mortality, patients were followed-up until death from PC, death from other causes, or December 31, 2013, whichever came first.

This study was approved by the Institutional Review Board of Seoul National University Hospital (IRB number: E-1612-007-809). The need for written informed consent from individual subjects was waived by the Institutional Review Board of Seoul National University Hospital, as we used de-identified data. All research was performed in accordance with relevant guidelines and regulations.

### Exposure to medication use

The primary exposure of interest was cumulative use of aspirin, statins, and metformin. Medication codes, dosage, duration, and the date of pharmacy claims data for the drugs were collected for medication use assessment. Exposure to each medication was considered to be a time-dependent risk factor. Follow-up time for each subject was divided into a time window of 2 years (Supplementary Fig. S1), and the cumulative use of each medication during the previous time window (i.e. previous 2 years) was calculated as exposure. Cumulative use of aspirin was calculated by summing the total prescription duration (number of days) from the time the prescription for aspirin was filled during each time window. For metformin and statins, the DDD system was used to calculate the cumulative defined daily dose (cDDD). The DDD system is recommended by the World Health Organization (WHO) for drug utilization studies as it provides the average maintenance dose of each medication according to its main indication in adults^33^. Cumulative DDD is the total sum of the DDDs for each individual medication representing total exposure for each individual during the study period. In this study, we summed all doses for the prescribed metformin and for statins every 2 years to obtain the cumulative exposure during each time window. For analyses, subjects were classified according to their medication use as follows: non-users, < 182.5, 182.5–365, 365–547.5, or 547.5–730 days of use (for aspirin) or cDDD (for metformin or statins) per 2-year time window. In addition, subjects who were ever prescribed with each drug during a 2-year time window were classified as “users of any amount” of the respective drug.

### Covariates

Data on baseline sociodemographic, clinical, and lifestyle characteristics of the study population which were available from the KNHI claims database were collected. For sociodemographic factors, age, BMI, and level of income (ranks 1–3 & Medicaid, ranks 4–6, ranks 7–10) were included. For clinical and lifestyle factors, Charlson comorbidity index (CCI) (0, 1–2, 3–4, or ≥ 5)^34,35^, smoking status (never, former, current), and alcohol consumption level (0–10, 10–20, 20–30, 30–40, ≥ 40 g/day) were included.

### Statistical analysis

Descriptive statistics were used to characterize the baseline characteristics of the study population. Cumulative duration of use of aspirin, and cDDD for statins and metformin use for each two-year time window during 2002–2011 were calculated. Proportion of concomitant use of aspirin, statins, and metformin was evaluated using kappa statistics.

To examine the associations between cumulative exposure to medications with PC incidence or mortality, time-dependent Cox proportional hazards models were used. First, we analyzed the associations of cumulative use of each medication with PC incidence or mortality without adjustment for concomitant use of other medications of interest. We then performed the same Cox proportional hazard regression adjusting for concomitant use of aspirin, statins, and metformin (e.g. aspirin use was adjusted for statins use and metformin use) to investigate the independent effects of each medication. Both models were adjusted for the potential confounders of age, income, BMI, smoking status, alcohol consumption, and CCI.

For analyses of metformin use, subjects were classified as diabetics (n = 24,944 in the baseline period) if they had a pharmacy claims record for any diabetic medication during each time window. Among them, subjects who had never used metformin during each time window were classified as non-metformin users. Non-diabetic subjects were used as a reference group for primary analyses (Table 2), and non-metformin users were used as a reference group for supplementary analyses (Supplementary Table S2).

Furthermore, a sensitivity analysis was performed to evaluate the effect of prevalence bias. Associations between cumulative exposure to medications with PC incidence or mortality were analyzed among new users of drugs (who were not using aspirin, statins, metformin, or other diabetic medications during 2002–2003). Exposure to medication use was assessed from January 1, 2004, and patients were followed-up from January 1, 2006 for this analysis.

In addition, to evaluate the long-term effect of drug use, we performed a secondary analysis using the usual Cox regression model with cumulative duration of drug use during 2002–2007 inserted as an exposure variable. Cumulative exposure to each drug was categorized as follows: < 2 years, 2–4 years, and 4–6 years. Patients were followed-up for PC incidence and mortality from January 1, 2008 in this analysis.

All analyses were performed using STATA version 14.1 (StataCorp, College Station, TX, USA); statistical significance was defined as a two-sided *p* value < 0.05.

## Results

### Cumulative use of aspirin, statins, and metformin

Table 1 shows the cumulative use of aspirin, statins, and metformin in 2-year time windows. For each medication, the number of users, duration of medication use, and cDDD of medication use increased over time. However, only a small proportion of subjects used these medications over the long-term or had a high cumulative dose. Concomitant use of the medications assessed by Cohen’s kappa value was highest for the aspirin-statins combination throughout the time windows (e.g. Cohen’s kappa value was 0.22 in the 2002–2003 period).

**Table 1:** Cumulative use of aspirin, statins, and metformin among the study population.

	2002–2003 (n = 388,760)	2004–2005 (n = 388,760)	2006–2007^a^ (n = 382,415)	2008–2009^a^ (n = 373,980)	2010–2011^a^ (n = 364,678)
**Aspirin**					
User of any amount, n (%)	32,716 (8.4)	52,518 (13.5)	64,085 (16.8)	63,295 (16.9)	81,322 (22.3)
*Duration of use per 2 years, days*					
< 182.5	16,726 (51.1)	20,941 (39.9)	21,028 (32.8)	18,896 (29.9)	20,367 (25.0)
182.5–365.0	5853 (17.9)	8315 (15.8)	14,105 (22.0)	7608 (12.0)	9480 (11.7)
365.0–547.5	3939 (12.04)	7196 (13.7)	8485 (13.24)	7598 (12.0)	10,011 (12.3)
≥ 547.5	6198 (18.94)	16,066 (30.6)	20,467 (31.9)	29,193 (46.1)	41,464 (51.0)
**Statins**					
User of any amount, n (%)	15,971 (4.1)	26,988 (6.9)	38,449 (10.1)	46,339 (12.4)	67,442 (18.5)
*cDDD per 2 years*					
< 182.5	13,476 (84.4)	18,983 (70.3)	22,924 (59.6)	23,210 (50.1)	27,647 (41.0)
182.5–365.0	2014 (12.6)	5322 (19.7)	8792 (22.9)	10,466 (22.6)	19,372 (28.7)
365.0–547.5	418 (2.6)	1693 (6.3)	3841 (10.0)	5493 (11.9)	8078 (12.0)
≥ 547.5	63 (0.4)	990 (3.7)	2892 (7.5)	7170 (15.5)	12,345 (18.3)
**Metformin**					
Non-DM	363,816 (93.6)	354,523 (91.2)	342,353 (89.5)	332,265 (88.9)	310,739 (85.2)
DM, Non-user (other drugs only), n (%)	11,545 (3.0)	13,676 (3.5)	14,090 (3.7)	11,951 (3.2)	10,438 (2.9)
DM, User of any amount, n (%)	13,399 (3.4)	20,561 (5.3)	25,972 (6.8)	29,764 (8.0)	43,501 (11.9)
*cDDD per 2 years*					
< 182.5	9370 (69.9)	12,431 (60.5)	15,362 (59.2)	15,143 (50.9)	19,916 (45.8)
182.5–365.0	2946 (22.0)	5312 (25.8)	6606 (25.4)	7319 (24.6)	11,936 (27.4)
365.0–547.5	812 (6.1)	1737 (8.4)	2036 (7.8)	2571 (8.6)	4400 (10.1)
≥ 547.5	271 (2.0)	1081 (5.3)	1968 (7.6)	4731 (15.9)	7249 (16.7)
**Kappa statistics, Kappa coefficient (proportion of agreement %)**					
Aspirin-Statin	0.22 (90.77%)	0.27 (86.51%)	0.32 (84.20%)	0.39 (84.67%)	0.39 (80.03%)
Statin-Metformin (all)	0.13 (90.22%)	0.17 (85.62%)	0.20 (83.00%)	0.26 (85.65%)	0.26 (78.67%)
Statin-Metformin (DM subjects)	0.12 (93.60%)	0.16 (90.38%)	0.21 (87.79%)	0.28 (86.84%)	0.28 (81.34%)
Aspirin-Metformin (all)	0.05 (50.69%)	0.06 (50.82%)	0.06 (51.21%)	0.08 (52.10%)	0.03 (50.47%)
Aspirin-Metformin (DM subjects)	0.02 (48.61%)	0.03 (46.07%)	0.05 (47.19%)	0.08 (48.78%)	0.05 (48.20%)

cDDD, cumulative defined daily dose; DM, diabetes mellitus.

^a^Total number was identified after excluding those who were diagnosed with cancers and those who died during follow-up.

### Baseline characteristics

Supplementary Table S1 presents the baseline characteristics of the study population by medication subgroups in the 2002–2003 period. Medication users were older, had a higher BMI, more comorbidities, consumed more alcohol, but smoked less than non-medication users.

### Associations of aspirin, statins, and metformin use with PC incidence and mortality

Over the mean follow-up of 9.5 years, 4518 PC cases and 486 deaths from PC were identified. Number of deaths from other causes was 41,455 during follow-up.

Table 2 shows the results of multivariate-adjusted analyses for associations of cumulative medication use with PC incidence and mortality. Use of any amount of aspirin was not associated with PC incidence (adjusted hazard ratio [aHR] 1.03, 95% CI 0.95–1.11, *p* value 0.505, adjusted for concomitant medications). This null association was consistent across different cumulative durations of aspirin use. Use of any amount of aspirin showed no significant relation to PC mortality, but the use of aspirin for ≥ 547.5 days (per 2-year interval) was associated with a marginal decrease in the risk of PC mortality (aHR 0.71, 95% CI 0.50–1.02, *p* value 0.062).

**Table 2:** Multivariate-adjusted^a^ analyses for associations of aspirin, statins, and metformin use with incidence and mortality of prostate cancer.

	Prostate cancer incidence		Prostate cancer mortality	
	Unadjusted for concomitant medication use	Adjusted for concomitant medication use^b^	Unadjusted for concomitant medication use	Adjusted for concomitant medication use^b^
	aHR (95% CI)	aHR (95% CI)	aHR (95% CI)	aHR (95% CI)
**Aspirin**				
Non-users	1.00 (Reference)	1.00 (Reference)	1.00 (Reference)	1.00 (Reference)
Users of any amount	1.02 (0.95 to 1.09)	1.03 (0.95 to 1.11)	1.00 (0.82 to 1.23)	0.97 (0.78 to 1.21)
*Duration of use per 2 years, days*				
< 182.5	1.04 (0.93 to 1.16)	1.04 (0.93 to 1.17)	1.24 (0.92 to 1.66)	1.12 (0.83 to 1.52)
182.5–365.0	0.97 (0.82 to 1.13)	0.98 (0.83 to 1.15)	1.08 (0.70 to 1.66)	0.99 (0.64 to 1.55)
365.0–547.5	1.06 (0.90 to 1.26)	1.08 (0.91 to 1.29)	1.30 (0.84 to 2.03)	1.21 (0.77 to 1.91)
≥ 547.5	1.01 (0.91 to 1.12)	1.03 (0.92 to 1.15)	**0.70 (0.50 to 0.98)**	**0.71 (0.50 to 1.02)**
*P* for trend	0.699	0.567	0.203	0.147
**Statins**				
Non-users	1.00 (Reference)	1.00 (Reference)	1.00 (Reference)	1.00 (Reference)
Users of any amount	1.03 (0.95 to 1.13)	1.05 (0.95 to 1.15)	1.06 (0.82 to 1.36)	1.04 (0.79 to 1.37)
*cDDD per 2 years*				
< 182.5	1.07 (0.95 to 1.20)	1.08 (0.96 to 1.21)	1.28 (0.93 to 1.76)	1.18 (0.85 to 1.64)
182.5–365.0	1.04 (0.89 to 1.23)	1.06 (0.90 to 1.25)	0.98 (0.61 to 1.57)	0.95 (0.59 to 1.56)
365.0–547.5	1.03 (0.82 to 1.31)	1.05 (0.82 to 1.34)	0.96 (0.47 to 1.94)	0.95 (0.46 to 1.94)
≥ 547.5	0.87 (0.67 to 1.12)	1.01 (0.77 to 1.32)	0.52 (0.21 to 1.26)	0.75 (0.31 to 1.85)
*P* for trend	0.927	0.851	0.470	0.426
**Metformin**				
Non-DM	1.00 (Reference)	1.00 (Reference)	1.00 (Reference)	1.00 (Reference)
DM, Non-users (other drugs only)	0.88 (0.76 to 1.03)	0.87 (0.75 to 1.01)	**1.99 (1.43 to 2.77)**	**2.01 (1.44 to 2.81)**
DM, Users of any amount	**0.87 (0.78 to 0.97)**	**0.86 (0.77 to 0.96)**	1.15 (0.87 to 1.53)	1.15 (0.86 to 1.54)
*cDDD per 2 years*				
< 182.5	**0.86 (0.75 to 0.99)**	**0.85 (0.73 to 0.98)**	1.32 (0.92 to 1.90)	1.32 (0.91 to 1.91)
182.5–365.0	1.01 (0.84 to 1.21)	0.99 (0.82 to 1.19)	**1.62 (1.03 to 2.56)**	**1.70 (1.07 to 2.69)**
365.0–547.5	0.84 (0.60 to 1.18)	0.82 (0.58 to 1.15)	0.99 (0.37 to 2.67)	1.05 (0.39 to 2.82)
≥ 547.5	**0.49 (0.32 to 0.75)**	**0.47 (0.30 to 0.74)**	§	§
*P* for trend^c^	0.357	0.617	0.008	0.027

aHR, adjusted hazard ratio; CI, confidence interval; cDDD, cumulative defined daily dose; DM, diabetes mellitus.

^a^Adjusted for age (5-year group), body mass index (continuous), income, Charlson comorbidity index (continuous), smoking status, and alcohol consumption.

^b^Additionally adjusted for concurrent use of aspirin, statins, and metformin.

^c^P for trend was calculated among diabetic patients only.

^§^Not calculated due to the low number (Number of prostate cancer-specific death case was 0 in this group).

Use of any amount of statins was not associated with either PC incidence or PC mortality, regardless of concomitant use of other medications or cumulative exposure to statins (aHR 1.05, 95% CI 0.95–1.15, *p* value 0.335, and aHR 1.04, 95% CI 0.79–1.37, *p* value 0.769, respectively).

Use of any amount of metformin was associated with reduced risk of PC incidence (aHR 0.86, 95% CI 0.77–0.96, *p* value 0.006) compared to that in non-diabetic patients. When examining the differences according to cDDD, those with metformin use of ≥ 547.5 cDDD showed a significantly decreased risk of PC incidence (aHR 0.47, 95% CI 0.30–0.74, *p* value 0.001) compared to non-diabetic patients. Metformin use of < 182.5 cDDD was also associated with lower incidence of PC (aHR 0.85, 95% CI 0.73–0.98, *p* value 0.023). For mortality, subjects with diabetes who were using anti-diabetic drugs other than metformin or using 182.5–365.0 cDDD of metformin had higher PC mortality than non-diabetic subjects (aHR 2.01, 95% CI 1.44–2.81, *p* value < 0.001, and aHR 1.70, 95% CI 1.07–2.69, *p* value 0.025, respectively). However, subjects with higher cumulative doses of metformin (365–547.5 cDDD) did not show increased PC mortality (aHR 1.05, 95% CI 0.39–2.82, *p* value 0.930). Overall, users of any amount of metformin showed no increase in PC mortality compared to non-diabetics (aHR 1.15, 95% CI 0.86–1.54, *p* value 0.353).

Supplementary Table S2 shows the results of analyses for metformin use with non-metformin users as a reference group. Metformin use < 547.5 cDDD was not associated with PC incidence compared to that of subjects using other diabetic drugs, whereas subjects using ≥ 547.5 cDDD of metformin showed lower PC incidence (aHR 0.54, 95% CI 0.34–0.87). For mortality, both non-diabetic subjects and users of any amount of metformin showed lower PC mortality than non-metformin users (aHR 0.50, 95% CI 0.36–0.70, and aHR 0.62, 95% CI 0.41–0.94, respectively).

Supplementary Table S3 shows the result of sensitivity analysis among new users of drugs, with similar trend to that observed in primary analysis. Use of aspirin for ≥ 547.5 days was associated with lower PC mortality among new users (aHR 0.51, 95% CI 0.26–0.98). Metformin use of ≥ 547.5 cDDD was associated with lower PC incidence compared to that of non-diabetic subjects (aHR 0.18, 95% CI 0.07–0.52). However, we could not determine the PC mortality risk among subjects with high cumulative doses of metformin (≥ 365 cDDD), because the number of PC-specific death in this group was zero among new users.

Supplementary Table S4 shows the result of usual Cox regression analysis with cumulative exposure to the drug during 2002–2007 as a risk factor. A similar trend to that of the main analysis was observed, although the statistical power was decreased. PC mortality risk was lower in subjects with aspirin use of 4–6 years than in non-users, yet statistically insignificant (aHR 0.76, 95% CI 0.35–1.64). Metformin users showed a marginal decrease in PC incidence compared to that in non-diabetics (aHR 0.77, 95% CI 0.54–1.10 in users for 4–6 years). However, use of metformin showed no definite association with PC mortality risk in this analysis.

## Discussion

In this population-based, longitudinal cohort study of 388,760 Korean men, we found that aspirin use was not associated with prostate cancer incidence. However, aspirin use of higher cumulative duration (≥ 547.5 days per 2 years) was associated with a 30% lower risk of PC mortality, although the relationship was statistically marginal. Our finding is consistent with a recent population-based cohort study conducted in the USA^36^ that found that aspirin use was not related to PC incidence, but inversely associated with PC mortality (HR 0.59, 95% CI 0.36–0.96). A possible explanation for the different impacts of aspirin on PC incidence and PC mortality is the potential therapeutic effects of aspirin on cancer. There is evidence that post-diagnosis aspirin use might reduce tumor metastasis and improve survival in cancer patients^37–39^. In a study of PC patients who received radical radiotherapy, aspirin use decreased early biochemical failure^37^. In another study of PC patients who received radical treatment, use of anticoagulants, mainly aspirin, reduced the distant metastasis rate compared with non-aspirin use patients (1% vs 5%; *p* = 0.0248)^38^. Aspirin might inhibit the growth of tumors and cell invasion^40,41^.

The protective effect of aspirin against PC mortality varied across cumulative duration of aspirin use. Cumulative use of aspirin for ≥ 547.5 days decreased the risk of PC mortality, while aspirin use for < 547.5 days showed no association with PC mortality. This implies the presence of a threshold effect of aspirin in cancer treatment. A similar result was observed in a nationwide cohort study of 29,136 PC patients in Denmark^11^; long-term use (≥ 1096 days) or a high cumulative amount (≥ 1096 tablets) of post-diagnosis aspirin reduced PC mortality (aHR 0.79, 95% CI 0.67–0.93, aHR 0.77, 95% CI 0.65–0.91, respectively), whereas short-term use or low cumulative use did not affect mortality. Another Irish cohort study also reported that patients who received a high dosing intensity of aspirin had a reduced risk of PC mortality (HR 0.73, 95% CI 0.51–1.05)^12^. Further research is needed to clarify whether aspirin has a threshold or dose–response effect on PC mortality.

As for statins, cumulative use of statins showed no association with the incidence or mortality of PC in our study. Despite multiple reports on the anti-cancer effects of statins, there are also conflicting results in the earlier literature regarding the association between statins and PC^16–19,22,42^. However, recent large-scale studies and a meta-analysis found no significant associations between statins use and PC incidence or mortality^22,43–46^, consistent with our findings in this study. One study reported that after adjusting for cholesterol level and prostate-specific antigen (PSA) screening rate, the protective effect of pre-diagnosis statins use on PC mortality disappeared, implying the healthy user effect^45^. To further elucidate the effects of statins, further studies should focus on identifying possible confounders.

In the current study, use of any amount of metformin was associated with lower PC incidence, and the degree of reduction was similar to that observed in a recent meta-analysis (aHR 0.91, 95% CI 0.85–0.97)^47^. Association of metformin use with PC incidence was strongest in diabetic subjects who used metformin for ≥ 547.5 cDDD per two years: PC incidence in this group was less than 50% of that in non-diabetic subjects (*p* = 0.001). This is in line with a Taiwanese cohort study that reported an inverse dose–response relationship between cumulative duration and dose of metformin with PC incidence (*p* trend < 0.001 in both)^14^. However, we observed a threshold effect rather than a dose–response relationship in our study.

Subjects with diabetes who were non-metformin users showed similar decrease in PC incidence to that of users of any amount of metformin in this study, though the result was not statistically significant (aHR 0.87, 95% CI 0.75–1.01). Previous studies found no significant difference in incidence of PC between subjects without diabetes and subjects with diabetes who were non-users of metformin^13,48^. According to results of our supplementary analysis, no difference was observed in risk of PC incidence among subjects using low cumulative doses of metformin (< 547.5 cDDD) compared to diabetic subjects using drugs other than metformin, whereas subjects using high cumulative doses of metformin (≥ 547.5 cDDD) showed lower PC incidence. It is possible that diabetes medications other than metformin decrease PC incidence risk, but metformin could have better protective effects than other drugs when used at high cumulative doses.

Subjects with diabetes who were not using metformin or used low cumulative doses (182.5–365.0 cDDD) had higher PC mortality than non-diabetic subjects. Similarly, previous studies have suggested that diabetes increases the risk of mortality in PC patients^49,50^. In a meta-analysis of 274,677 patients, diabetes was associated with a 29% increased risk of PC mortality (relative risk 1.29, CI 1.22–1.38)^49^. Suggested mechanisms of worse cancer prognosis in diabetic patients include exacerbated progression of cancer induced by steroidogenesis and diminished response to radiotherapy^51,52^.

Meanwhile, diabetic subjects using metformin with a longer cumulative dose (365.0–547.5 cDDD) did not show an increase in risk of PC mortality compared to non-diabetic subjects, suggesting that metformin had a protective effect. Multiple studies have reported that metformin might reduce the recurrence, mortality, and development of castration-resistant cancer among PC patients^15,47,53^. In a retrospective cohort study of diabetic patients with PC, cumulative duration of post-diagnosis metformin showed a linear inverse relationship to PC-specific mortality^15^. A 24% reduction in PC mortality was observed for each additional 6 months of metformin treatment (aHR 0.76, 95% CI 0.64–0.89). However, it is unclear whether metformin had a threshold or dose–response effect on PC mortality in our study.

Our findings suggest that compliance to certain cardiovascular medications might associate with PC incidence and mortality. For adults who are already taking aspirin or metformin, regular use of these medications could be related to reduced risk of PC mortality. For adults who are taking metformin, its use at a higher cumulative dose might be associated with reduced PC incidence. Further studies are warranted to explore cut-off values of these medications for favorable effects.

A strength of our study is our large sample representative of the Asian population (n = 388,760). Furthermore, we performed simultaneous examinations of the independent effects of common cardiovascular medications with adjustment for cumulative use of other medications. Our study also had several limitations; first, we identified cancer cases using disease codes and reimbursement data for medical services, thus the incidence of cancer may have been slightly underreported in our study. Very old patients who refused treatment or surveillance would have been omitted, and patients under watchful waiting or active surveillance were not included, which might also have affected in the prognosis of PC cases observed in the analysis. Also, PC patients who chose not to receive active treatment might be more likely to have comorbidities, including diabetes mellitus and heart disease, and to use cardiovascular medications than patients who underwent active treatment, which might have resulted in overestimated effects of these medications for PC prevention. However, such an effect was thought to be weak because of the small proportion of PC patients who do not receive active treatment in Korea^54^. Second, there may have been detection bias. Patients taking aspirin, statins, or metformin may visit medical facilities more frequently than those not taking these medications, and therefore have a higher likelihood of undergoing cancer screening and being diagnosed with PC. If this detection bias were present, however, the actual associations would be stronger than our current estimates. Also, prevalence bias might exist as we included the subjects who were prevalent users of cardiovascular drugs when follow-up began. Nevertheless, the results of sensitivity analyses among new users of drugs were similar to those of primary analysis, implying low risk of such bias and robustness of our study results. Third, since study participants were limited to those who underwent national health screening, the result of this study might not be generalizable. Study subjects could have visited a medical facility more often than the general population, producing a higher detection rate of PC. Fourth, because low-dose aspirin is an over-the-counter drug in Korea, the use of aspirin could have been underestimated. Finally, as we used administrative data, specific information on disease status, such as cancer stage or serum PSA level, which is a well-known marker of the biochemical control rate of PC, are lacking.

In conclusion, metformin use was associated with lower PC incidence in the general male population in Korea. Use of aspirin and that of metformin among diabetic patients were independently associated with lower PC mortality, while use of statins was not significantly associated with either PC incidence or mortality. Further research is required to provide evidence of the anti-cancer effects of aspirin and metformin.

## Supplementary Information

Supplementary Figure.

Supplementary Tables.

## Data Availability

The data that support the findings of this study are available from NHIS. Restrictions apply to the availability of these data, which were used under license for this study. Data are available from the authors with the permission of NHIS.
