# Supplementary figures and images for: Population-wide impacts of aspirin, statins, and metformin use on prostate cancer incidence and mortality
Source: Sci Rep. 2021 Aug 9;11:16171. doi: 10.1038/s41598-021-95764-3 (PMC8352896; doi:10.1038/s41598-021-95764-3)

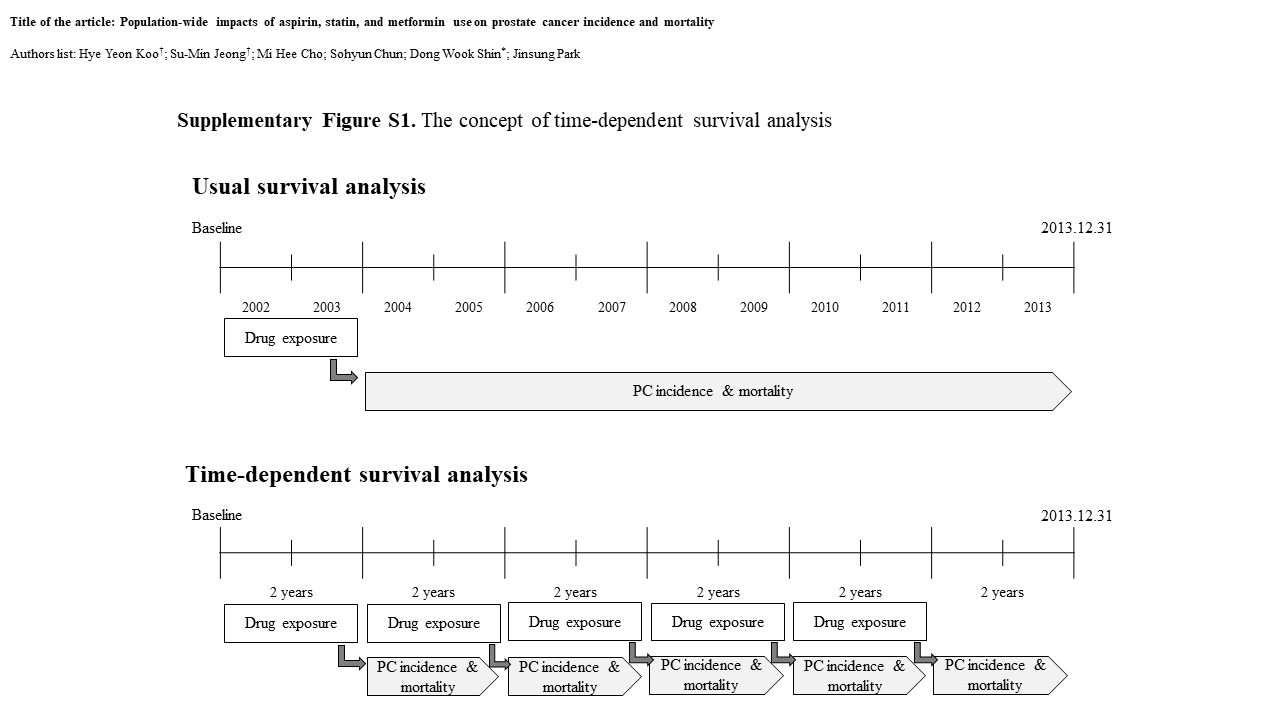

Supplement: Supplementary file 1 — Supplementary Figure. [file 41598_2021_95764_MOESM1_ESM.tif]
