# Supplementary material for: Population-wide impacts of aspirin, statins, and metformin use on prostate cancer incidence and mortality
Source: Sci Rep. 2021 Aug 9;11:16171. doi: 10.1038/s41598-021-95764-3 (PMC8352896; doi:10.1038/s41598-021-95764-3)
Supplement: Supplementary file 2 — Supplementary Tables. [file 41598_2021_95764_MOESM2_ESM.docx]

**Title of the article: Population-wide impacts of aspirin, statins, and metformin use on prostate cancer incidence and mortality**

Authors list: Hye Yeon Koo^†^; Su-Min Jeong^†^; Mi Hee Cho; Sohyun Chun; Dong Wook Shin^*^; Jinsung Park

|  | Total  (n=388,760) | Aspirin | | | Statins | | | Metformin | | | |
| --- | --- | --- | --- | --- | --- | --- | --- | --- | --- | --- | --- |
|  |  | Non-user  (n=356,044) | User  (n=32,716) |  | Non-user  (n=372,789) | User  (n=15,971) |  | Non-DM  (n=363,816) | DM,  Non-user (n=13,399) | DM,  user (n=11,545) |  |
| Age, years, n (%) |  |  |  |  |  |  |  |  |  |  |  |
| 40 – 49 | 163,684 (42.1) | 15,8733(44.6) | 4,951 (15.1) |  | 159,198 (42.7) | 4,486 (28.1) |  | 158,657 (43.6) | 2,755 (20.6) | 2,272 (19.7) |  |
| 50 – 59 | 92,019 (23.7) | 84,820 (23.8) | 7,199 (22.0) |  | 87,730 (23.5) | 4,289 (26.9) |  | 85,732 (23.6) | 3,398 (25.4) | 2,889 (25.0) |  |
| 60 – 69 | 104,196 (26.8) | 89,163 (25.0) | 15,033 (45.9) |  | 98,399 (26.4) | 5,797 (36.3) |  | 93,475 (25.7) | 5,724 (42.7) | 4,997 (43.3) |  |
| 70 – 79 | 28,861 (7.4) | 23,328 (6.6) | 5,533 (16.9) |  | 27,462 (7.4) | 1,399 (8.8) |  | 25,952 (7.1) | 1,522 (11.4) | 1,387 (12.0) |  |
| BMI, kg/m^2^, mean (SD) | 21.9 (6.8) | 21.7 (7.0) | 24.0 (4.5) |  | 21.8 (6.9) | 24.4 (4.5) |  | 21.8 (6.9) | 23.9 (4.6) | 23.8 (4.7) |  |
| Income, n (%) |  |  |  |  |  |  |  |  |  |  |  |
| Rank 1-3 & Medicaid | 184,486 (47.5) | 169,089 (47.5) | 15,397 (47.1) |  | 176,267 (47.3) | 8,219 (51.5) |  | 173,102 (47.6) | 6,043 (45.1) | 5,341 (46.3) |  |
| Rank 4-6 | 101,277 (26.1) | 93,466 (26.3) | 7,811 (23.9) |  | 97,388 (26.1) | 3,889 (24.4) |  | 95,010 (26.1) | 3,383 (25.2) | 2,884 (25.0) |  |
| Rank 7-10 | 102,997 (26.5) | 93,489 (26.3) | 9,508 (29.1) |  | 99,134 (26.6) | 3,863 (24.2) |  | 95,704 (26.3) | 3,973 (29.7) | 3,320 (28.8) |  |

Supplementary Table S1. Baseline characteristics of the study population by medication use in 2002—2003*.

| CCI score, mean (SD) |  |  |  |  |  |  |  |  |  |  |  |
| --- | --- | --- | --- | --- | --- | --- | --- | --- | --- | --- | --- |
| 0 | 211,251 (54.3) | 203,274 (57.1) | 7,977 (24.4) |  | 207,818 (55.7) | 3,433 (21.5) |  | 206,674 (56.8) | 2,226 (16.6) | 2,351 (20.4) |  |
| 1-2 | 160,010 (41.2) | 140,855 (39.6) | 19,155 (58.5) |  | 150,143 (40.3) | 9,867 (61.8) |  | 146,348 (40.2) | 7,228 (53.9) | 6,434 (55.7) |  |
| 3-4 | 16,275 (4.2) | 11,276 (3.2) | 4,999 (15.3) |  | 13,873 (3.7) | 2,402 (15.0) |  | 10,239 (2.8) | 3,579 (26.7) | 2,457 (21.3) |  |
| ≥5 | 1,224 (0.3) | 639 (0.2) | 585 (1.8) |  | 955 (0.3) | 269 (1.7) |  | 555 (0.2) | 366 (2.7) | 303 (2.6) |  |
| Smoking status, n (%) |  |  |  |  |  |  |  |  |  |  |  |
| Never | 184,670 (47.5) | 166,401 (46.7) | 18,269 (55.8) |  | 176,471 (47.3) | 8,199 (51.3) |  | 171,643 (47.2) | 6,835 (51.0) | 6,192 (53.6) |  |
| Former | 52,495 (13.5) | 47,417 (13.3) | 5,078 (15.5) |  | 49,786 (13.4) | 2,709 (17.0) |  | 49,042 (13.5) | 1,814 (13.5) | 1,639 (14.2) |  |
| Current | 151,595 (39.0) | 142,226 (39.9) | 9,369 (28.6) |  | 146,532 (39.3) | 5,063 (31.7) |  | 143,131 (39.3) | 4,750 (35.5) | 3,714 (32.2) |  |
| Alcohol, g/day, n (%) |  |  |  |  |  |  |  |  |  |  |  |
| 0 – 10 | 256,467 (66.0) | 232,638 (65.3) | 23,829 (72.8) |  | 245,303 (65.8) | 11,164 (69.9) |  | 238,705 (65.6) | 9,587 (71.6) | 8,175 (70.8) |  |
| 10 – 20 | 71,103 (18.3) | 66,423 (18.7) | 4,680 (14.3) |  | 68,536 (18.4) | 2,567 (16.1) |  | 67,488 (18.6) | 1,926 (14.4) | 1,689 (14.6) |  |
| 20 – 30 | 5,488 (1.4) | 5,164 (1.5) | 324 (1.0) |  | 5,278 (1.4) | 210 (1.3) |  | 5,134 (1.4) | 200 (1.5) | 154 (1.3) |  |
| 30 – 40 | 22,604 (5.8) | 21,036 (5.9) | 1,568 (4.8) |  | 21,791 (5.8) | 813 (5.1) |  | 21,361 (5.9) | 646 (4.8) | 597 (5.2) |  |
| ≥ 40 | 33,098 (8.5) | 30,783 (8.6) | 2,315 (7.1) |  | 31,881 (8.6) | 1,217 (7.6) |  | 31,128 (8.6) | 1,040 (7.8) | 930 (8.1) |  |
| DM, diabetes mellitus; BMI, body mass index; CCI, Charlson comorbidity index.  *Categorical variables were compared using the chi-square test, and continuous variables were compared using Student's t-test (aspirin or statin) or ANOVA (metformin). P values for all comparisons were <0.001. | | | | | | | | | | | |

Supplementary Table S2. Multivariate-adjusted^a^ analyses for associations of metformin use with incidence and mortality of prostate cancer (with non-metformin users as a reference group)

|  | **Prostate cancer incidence** | | | | **Prostate cancer mortality** | | | |
| --- | --- | --- | --- | --- | --- | --- | --- | --- |
|  | Unadjusted for  concomitant medication use | | Adjusted for  concomitant medication use^b^ | | Unadjusted for  concomitant medication use | | Adjusted for  concomitant medication use^b^ | |
|  | aHR (95% CI) |  | aHR (95% CI) |  | aHR (95% CI) |  | aHR (95% CI) |  |
| **Case, N (%)** | 4,518 | | |  | 486 | | |  |
| **Cumulative use of metformin** |  |  |  |  |  |  |  |  |
| DM, Non-users (other drugs only) | 1.00 (Reference) |  | 1.00 (Reference) |  | 1.00 (Reference) |  | 1.00 (Reference) |  |
| Non-DM | 1.13 (0.98 to 1.32) |  | 1.15 (0.99 to 1.34) |  | **0.50 (0.36 to 0.70)** |  | **0.50 (0.36 to 0.70)** |  |
| DM, Users of any amount | 0.98 (0.82 to 1.17) |  | 0.97 (0.81 to 1.16) |  | **0.62 (0.41 to 0.93)** |  | **0.62 (0.41 to 0.94)** |  |
| cDDD per 2 years |  |  |  |  |  |  |  |  |
| <182.5 | 0.98 (0.80 to 1.19) |  | 0.97 (0.80 to 1.19) |  | 0.67 (0.42 to 1.06) |  | 0.66 (0.41 to 1.05) |  |
| 182.5 - 365.0 | 1.14 (0.91 to 1.44) |  | 1.13 (0.90 to 1.43) |  | 0.82 (0.48 to 1.40) |  | 0.84 (0.49 to 1.45) |  |
| 365.0 - 547.5 | 0.95 (0.66 to 1.37) |  | 0.94 (0.65 to 1.36) |  | 0.50 (0.18 to 1.40) |  | 0.52 (0.19 to 1.46) |  |
| ≥547.5 | **0.55 (0.35 to 0.87)** |  | **0.54 (0.34 to 0.87)** |  | § |  | § |  |
| *P* for trend^c^ | 0.357 |  | 0.414 |  | 0.008 |  | 0.018 |  |
| aHR, adjusted hazard ratio; CI, confidence interval; cDDD, cumulative defined daily dose; DM, diabetes mellitus  ^a^Adjusted for age (5-year group), body mass index (continuous), income, Charlson comorbidity index (continuous), smoking status, and alcohol consumption  ^b^Additionally adjusted for concurrent use of aspirin, statin, and metformin  ^c^P for trend was calculated among diabetic patients only.  §Not calculated due to the low number (Number of prostate cancer-specific death case was 0 in this group). | | | | | | | | |

Supplementary table S3. Associations of aspirin, statins, and metformin use with incidence and mortality of prostate cancer among new users of drugs (n=326,185) ^a^

|  | **Prostate cancer incidence** | | | | **Prostate cancer mortality** | | | |
| --- | --- | --- | --- | --- | --- | --- | --- | --- |
|  | Unadjusted for  concomitant medication use | | Adjusted for  concomitant medication use^b^ | | Unadjusted for  concomitant medication use | | Adjusted for  concomitant medication use^b^ | |
|  | aHR (95% CI) |  | aHR (95% CI) |  | aHR (95% CI) |  | aHR (95% CI) |  |
| **Aspirin** |  |  |  |  |  |  |  |  |
| Non-users | 1.00 (Reference) |  | 1.00 (Reference) |  | 1.00 (Reference) |  | 1.00 (Reference) |  |
| Users of any amount | 0.10 (0.90 - 1.10) |  | 0.99 (0.89 to 1.10) |  | 1.11 (0.822 to 1.486) |  | 1.12 (0.82 to 1.53) |  |
| Duration of use per 2 years, days |  |  |  |  |  |  |  |  |
| <182.5 | 1.04 (0.90 to 1.21) |  | 1.041 (0.90 to 1.21) |  | 1.33 (0.89 to 2.00) |  | 1.30 (0.86 to 1.97) |  |
| 182.5 - 365.0 | 1.05 (0.84 to 1.31) |  | 1.022 (0.81 to 1.28) |  | 1.69 (0.96 to 2.98) |  | 1.62 (0.91 to 2.89) |  |
| 365.0 - 547.5 | 0.99 (0.76 to 1.27) |  | 0.984 (0.76 to 1.28) |  | 1.27 (0.63 to 2.58) |  | 1.22 (0.59 to 2.54) |  |
| ≥547.5 | 0.92 (0.78 to 1.08) |  | 0.945 (0.79 to 1.13) |  | 0.57 (0.31 to 1.05) |  | 0.51 (0.26 to 0.98) |  |
| *P* for trend | 0.478 |  | 0.590 |  | 0.471 |  | 0.226 |  |
| **Statins** |  |  |  |  |  |  |  |  |
| Non-users | 1.00 (Reference) |  | 1.00 (Reference) |  | 1.00 (Reference) |  | 1.00 (Reference) |  |
| Users of any amount | 1.06 (0.94 to 1.20) |  | 1.10 (0.97 to 1.25) |  | 0.97 (0.66 to 1.44) |  | 0.90 (0.59 to 1.38) |  |
| cDDD per 2 years |  |  |  |  |  |  |  |  |
| < 182.5 | 1.09 (0.93 to 1.27) |  | 1.10 (0.94 to 1.29) |  | 0.73 (0.40 to 1.35) |  | 0.63 (0.34 to 1.18) |  |
| 182.5 - 365.0 | 1.12 (0.88 to 1.41) |  | 1.16 (0.91 to 1.47) |  | 1.11 (0.54 to 2.25) |  | 1.06 (0.51 to 2.20) |  |
| 365.0 - 547.5 | 1.24 (0.88 to 1.74) |  | 1.30 (0.92 to 1.84) |  | 2.03 (0.90 to 4.59) |  | 2.00 (0.86 to 4.62) |  |
| ≥ 547.5 | 0.74 (0.51 to 1.06) |  | 1.17 (0.79 to 1.75) |  | 0.87 (0.32 to 2.35) |  | 1.65 (0.59 to 4.57) |  |
| *P* for trend | 0.93 |  | 0.275 |  | 0.7 |  | 0.855 |  |
| **Metformin** |  |  |  |  |  |  |  |  |
| Non-DM | 1.00 (Reference) |  | 1.00 (Reference) |  | 1.00 (Reference) |  | 1.00 (Reference) |  |
| DM, Non-users (other drugs only) | 0.77 (0.58 to 1.02) |  | 0.750 (0.56 to 1.00) |  | 2.67 (1.61 to 4.45) |  | 2.61 (1.56 to 4.37) |  |
| DM, Users of any amount | 0.81 (0.67 to 0.98) |  | 0.787 (0.65 to 0.96) |  | 1.16 (0.69 to 1.93) |  | 1.15 (0.68 to 1.95) |  |
| cDDD per 2 years |  |  |  |  |  |  |  |  |
| <182.5 | 0.91 (0.72 to 1.14) |  | 0.88 (0.70 to 1.11) |  | 1.62 (0.92 to 2.84) |  | 1.61 (0.91 to 2.84) |  |
| 182.5 - 365.0 | 0.88 (0.58 to 1.33) |  | 0.85 (0.56 to 1.28) |  | 1.18 (0.38 to 3.71) |  | 1.19 (0.38 to 3.77) |  |
| 365.0 - 547.5 | 0.96 (0.43 to 2.15) |  | 0.92 (0.41 to 2.04) |  | § |  | § |  |
| ≥547.5 | 0.21 (0.08 to 0.55) |  | 0.18 (0.07 to 0.52) |  | § |  | § |  |
| *P* for trend^c^ | 0.002 |  | 0.001 |  | 0.003 |  | 0.002 |  |

aHR, adjusted hazard ratio; CI, confidence interval; cDDD, cumulative defined daily dose; DM, diabetes mellitus

^a^Adjusted for age (5-year group), body mass index (continuous), income, Charlson comorbidity index (continuous), smoking status, and alcohol consumption

^b^Additionally adjusted for concurrent use of aspirin, statin, and metformin

^c^P for trend was calculated among diabetic patients only.

§Not calculated due to the low number (Number of prostate cancer-specific death case was 0 in this group).

Supplementary Table S4. Usual Cox regression analysis for association of cumulative exposure to drugs (2002-2007) with incidence and mortality of prostate cancer

|  | **Prostate cancer incidence** | | | | **Prostate cancer mortality** | | | |
| --- | --- | --- | --- | --- | --- | --- | --- | --- |
|  | Unadjusted for  concomitant medication use | | Adjusted for  concomitant medication use^b^ | | Unadjusted for  concomitant medication use | | Adjusted for  concomitant medication use^b^ | |
|  | aHR (95% CI) |  | aHR (95% CI) |  | aHR (95% CI) |  | aHR (95% CI) |  |
| **Aspirin** |  |  |  |  |  |  |  |  |
| Non-users | 1.00 (Reference) |  | 1.00 (Reference) |  | 1.00 (Reference) |  | 1.00 (Reference) |  |
| Aspirin use |  |  |  |  |  |  |  |  |
| < 2 years | 1.07 (0.98 to 1.18) |  | 1.06 (0.96 to 1.17) |  | 1.39 (1.04 to 1.87) |  | 1.39 (1.02 to 1.88) |  |
| 2 – 4 years | 1.07 (0.93 to 1.24) |  | 1.06 (0.91 to 1.23) |  | 1.32 (0.84 to 2.07) |  | 1.29 (0.81 to 2.05) |  |
| 4 – 6 years | 1.11 (0.93 to 1.33) |  | 1.10 (0.91 to 1.33) |  | 0.70 (0.32 to 1.50) |  | 0.76 (0.35 to 1.64) |  |
| *P* for trend | 0.091 |  | 0.224 |  | 0.474 |  | 0.881 |  |
| **Statins** |  |  |  |  |  |  |  |  |
| Non-users | 1.00 (Reference) |  | 1.00 (Reference) |  | 1.00 (Reference) |  | 1.00 (Reference) |  |
| Statin use |  |  |  |  |  |  |  |  |
| < 2 years | 1.14 (1.03 to 1.27) |  | 1.15 (1.03 to 1.28) |  | 1.16 (0.81 to 1.66) |  | 1.07 (0.74 to 1.55) |  |
| 2 – 4 years | 1.15 (0.92 to 1.44) |  | 1.15 (0.91 to 1.46) |  | 1.26 (0.59 to 2.70) |  | 1.21 (0.55 to 2.63) |  |
| 4 – 6 years | 1.10 (0.75 to 1.61) |  | 1.11 (0.75 to 1.65) |  | NA |  | NA |  |
| *P* for trend | 0.018 |  | 0.033 |  | 0.905 |  | 0.499 |  |
| **Metformin** |  |  |  |  |  |  |  |  |
| Non-DM | 1.00 (Reference) |  | 1.00 (Reference) |  | 1.00 (Reference) |  | 1.00 (Reference) |  |
| DM, non-users (other drugs only) | 0.93 (0.73 to 1.18) |  | 0.91 (0.67 to 1.23) |  | 1.27 (0.52 to 3.12) |  | 1.29 (0.53 to 3.18) |  |
| Metformin use |  |  |  |  |  |  |  |  |
| < 2 years | 0.83 (0.71 to 0.99) |  | 0.80 (0.67 to 0.94) |  | 0.93 (0.54 to 1.60) |  | 0.86 (0.49 to 1.49) |  |
| 2 – 4 years | 0.86 (0.68 to 1.08) |  | 0.82 (0.65 to 1.03) |  | 1.11 (0.54 to 2.27) |  | 1.04 (0.50 to 2.13) |  |
| 4 – 6 years | 0.81 (0.57 to 1.16) |  | 0.77 (0.54 to 1.10) |  | 0.98 (0.31 to 3.08) |  | 1.08 (0.34 to 3.44) |  |
| *P* for trend | 0.054 |  | 0.016 |  | 0.694 |  | 0.881 |  |

NA: Non-applicable because there was no event case
